# Lineage-specific sequence evolution and exon edge conservation partially explain the relationship between evolutionary rate and expression level in *A. thaliana*

**DOI:** 10.1111/mec.13221

**Published:** 2015-06-05

**Authors:** Stephen J Bush, Paula X Kover, Araxi O Urrutia

**Affiliations:** Department of Biology and Biochemistry, University of BathBath, BA2 7AY, UK

**Keywords:** dN/dS, *Arabidopsis thaliana*, lineage-specific evolution, splice enhancer

## Abstract

Rapidly evolving proteins can aid the identification of genes underlying phenotypic adaptation across taxa, but functional and structural elements of genes can also affect evolutionary rates. In plants, the ‘edges’ of exons, flanking intron junctions, are known to contain splice enhancers and to have a higher degree of conservation compared to the remainder of the coding region. However, the extent to which these regions may be masking indicators of positive selection or account for the relationship between dN/dS and other genomic parameters is unclear. We investigate the effects of exon edge conservation on the relationship of dN/dS to various sequence characteristics and gene expression parameters in the model plant *Arabidopsis thaliana*. We also obtain lineage-specific dN/dS estimates, making use of the recently sequenced genome of *Thellungiella parvula*, the second closest sequenced relative after the sister species *Arabidopsis lyrata*. Overall, we find that the effect of exon edge conservation, as well as the use of lineage-specific substitution estimates, upon dN/dS ratios partly explains the relationship between the rates of protein evolution and expression level. Furthermore, the removal of exon edges shifts dN/dS estimates upwards, increasing the proportion of genes potentially under adaptive selection. We conclude that lineage-specific substitutions and exon edge conservation have an important effect on dN/dS ratios and should be considered when assessing their relationship with other genomic parameters.

## Introduction

Rates of sequence evolution are known to vary between genes, particularly at non-synonymous sites (Bromham 2009). Various genomic parameters are significant predictors of dN/dS, an estimate of the rate of protein evolution corrected by the underlying rate of substitution at synonymous sites. In a substantial number of species, including *Arabidopsis thaliana*, expression level is considered the best predictor of dN/dS ratios (Akashi 2003; Krylov *et al*. 2003; Wright *et al*. 2004; Drummond *et al*. 2005; Cherry 2010a), alongside expression breadth (an estimate of the proportion of tissues in which a gene is expressed) (Duret & Mouchiroud 2000; Winter *et al*. 2004; Zhang & Li 2004; Park & Choi 2010). Other variables, including codon usage bias (Urrutia & Hurst 2001; Xia *et al*. 2009), GC content (Ticher & Graur 1989; Cherry 2010b), protein multi-functionality (Hahn & Kern 2005; Podder *et al*. 2009), the number of interacting partners per protein (Fraser & Hirsh 2004; Makino & Gojobori 2006; Wang & Lercher 2011), recombination rate (Pál *et al*. 2001; Wright *et al*. 2006), gene/protein length (Coghlan & Wolfe 2000; Urrutia & Hurst 2003; Lemos *et al*. 2005; Stoletzki & Eyre-Walker 2007) and both intron number and length (Seoighe *et al*. 2005; Tang *et al*. 2006; Larracuente *et al*. 2008) have all been associated with dN/dS ratios.

Variations in dN/dS are thought to stem primarily from gene-specific selective pressures related to the functionality of their protein products (Tennessen 2008). As such, dN/dS is often used to identify those genes likely to be involved in adaptation (Yang & Bielawski 2000; Hurst 2002; Nielsen 2005). Determining which genes are under selection is important for understanding how genetic diversity is maintained and the relative importance of opposing selective forces in shaping a species’ genetic diversity.

As with many species, plant genes predominantly evolve under purifying selection (Gossmann *et al*. 2010), with low estimates for the number of positively selected genes in sorghum (Hamblin *et al*. 2006), maize (Ross-Ibarra *et al*. 2009), *A. thaliana* (Schmid *et al*. 2005; Slotte *et al*. 2011) and *A. lyrata* (Foxe *et al*. 2008). In these studies, dN/dS was calculated from pairwise alignments resulting in ratios which are a composite of substitutions in both lineages compared since their divergence from their last common ancestor. Using an outgroup species allows the calculation of lineage-specific dN/dS (Arbiza *et al*. 2006; Bakewell *et al*. 2007; Kawahara & Imanishi 2007; Weedall *et al*. 2008; Parmakelis *et al*. 2010; Toll-Riera *et al*. 2011) which could unmask further genes with species-specific signatures of positive selection and/or potentially stronger associations between certain genomic characteristics and the rate of sequence evolution. The model plant *Arabidopsis thaliana* is an ideal organism for investigating genomewide signatures of selection in the plant taxa as a sister species, *Arabidopsis lyrata* (with an estimated 13 mya divergence time from *A. thaliana*), has been sequenced (Hu *et al*. 2011). *Thellungiella parvula* [43 mya divergence from *A. thaliana* (Dassanayake *et al*. 2011)] provides a suitable outgroup for assessing lineage-specific sequence evolution. The availability of multiple *A. thaliana* genomes (Cao *et al*. 2011; Gan *et al*. 2011) enables the assessment of intraspecific diversity, which can be used to estimate deviations from a neutral expectation based on both sequence divergence and intraspecific variation, such as the neutrality index (NI) (Haldane 1956).

The interpretation of dN/dS and NI estimates assumes that synonymous substitutions are mostly evolving under neutral or nearly neutral conditions and are a proxy of the underlying mutation rate. However, exon sequences can contain exonic splicing enhancers (ESEs), sequence motifs involved in both constitutive and regulated splicing by facilitating the assembly of splicing complexes (Tacke & Manley 1999; Blencowe 2000; Zheng 2004). ESEs are enriched in the vicinity of splice sites, particularly downstream of a splice acceptor, with their peak abundance increasing closer to an exon–intron boundary (Wu *et al*. 2005). As higher conservation in this region, including at synonymous sites, can reflect differential patterns of codon usage (Comeron & Guthrie 2005; Parmley & Hurst 2007; Warnecke & Hurst 2007; Caceres & Hurst 2013) and affect the overall dN/dS estimate per gene (Carlini & Genut 2006; Parmley *et al*. 2006), this may influence relationships between dN/dS and various genomic parameters, particularly in compact, intron-rich genomes. In *A. thaliana*'s genome, 75% of the genes are multi-exonic, 29% of the exons are below 100 bp, the median exon length is 53 codons, and ESE hexamers have been identified (Pertea *et al*. 2007). Thus, ESE conservation could have a strong impact on estimates of dN/dS, and consequently on estimates of the relative contribution of positive and purifying selection to *A. thaliana* genome evolution.

It is not yet known, however, how dN/dS estimates in plants are influenced either by increased conservation at exon edges or by the introduction of an outgroup species to obtain lineage-specific estimates nor how this may affect the covariance between the rate of sequence evolution and any genomic parameter previously shown to be a significant predictor of NI and/or dN/dS.

Here, we address this issue by examining coding sequence evolution in *A. thaliana*, with *A. lyrata* and *T. parvula* as comparison species. We investigate whether the calculation of lineage-specific sequence evolutionary rate and/or the removal of exon edges (i) may unmask a larger proportion of genes with signatures of selection, (ii) alter the relationship between expression level and evolutionary rate and (iii) alter the association between dN/dS and other structural and functional parameters previously identified as dN/dS correlates in one or more other species.

## Materials and methods

### Genome sequences and gene annotations

Exon coordinates for *A. thaliana* strain Col-0 were obtained from The Arabidopsis Information Resource (TAIR) (http://ftp://ftp.arabidopsis.org/, file ‘TAIR10_exon_20101028’, downloaded 15 February 2013). The *A. lyrata* genome (Hu *et al*. 2011), strain MN47 (Entrez genome project ID 41137), was obtained from GenBank (http://www.ncbi.nlm.nih.gov/nuccore/ADBK00000000, downloaded 17 October 2012). The *T. parvula* genome, version 2.0 (Dassanayake *et al*. 2011), was obtained from http://thellungiella.org/blast/db/TpV8-4.fa (downloa-ded 17 October 2012).

### Other data sources

Codon usage bias per gene was expressed both as the effective number of codons (ENC) (Wright 1990) and as the frequency of optimal codons (*F*_op_) (Ikemura 1981). The number of protein–protein interactions (PPIs) per gene was obtained from BioGRID, version 3.1.75 (Stark *et al*. 2006, 2011). Recombination data were obtained from Marais *et al*. (2004); this variable is used as a control as an insignificant relationship between recombination and dN/dS is expected in an effectively obligate selfer. A gene's degree of multi-functionality was measured as the number of GOslim terms assigned to it for biological processes. ‘GOslim’ is a condensed set of gene ontology (GO) categories, obtained from TAIR (http://ftp://ftp.arabidopsis.org/home/tair/Ontologies/Gene_Ontology/ATH_GO_GOSLIM.txt, downloaded 8 October 2013) (Berardini *et al*. 2004). The majority of GOslim terms (∼87%) are derived from curated experimental or computational evidence, rather than being inferred from sequence similarity, which can result in higher false prediction rates (Jones *et al*. 2007). All raw data used in this study are available in Table S1 (Supporting information).

### Tests of sequence evolution and selection

Two measures of the degree and direction to which *A. thaliana* sequences diverge from a neutral expectation were calculated – a neutrality index (NI) and dN/dS. Calculations require data on the number of polymorphic and diverged residues in each sequence. To obtain the former, we used single nucleotide polymorphism (SNP) data obtained after aligning 17 fully sequenced and independently assembled accessions against the Col-0 reference genome (Gan *et al*. 2011) (data from Po-0 were not used as it has both unusually high heterozygosity and similarity to Oy-0). Diverged positions were identified from pairwise alignments of *A. thaliana* against both *A. lyrata* and *T. parvula*. Alignments were made for 21 198 genes against *A. lyrata* and 10 289 genes against *T. parvula*, of which 7086 genes could be aligned against both. Alignments were first obtained for exons in the longest available transcript per *A. thaliana* gene, using blastn (Altschul *et al*. 1990) with default parameters and a significance threshold of 1e^−10^. These were refined by applying the Smith–Waterman algorithm to the best blastn hit [fasta36.3.5d with parameters –A –a (require alignments to use entire sequence)] (Pearson 2000). The resulting alignments were then concatenated to create a single sequence alignment per gene. To ensure the alignment was in-frame, the translated *A. thaliana* sequence was aligned against either the *A. lyrata* or *T. parvula* sequence using tblastn (default parameters and significance threshold 1e^−10^).

For genes with at least 150 aligned bases, dN/dS was estimated from the concatenated sequences using the Yang and Nielson model, as implemented in the yn00 package of PAML (Yang 2007). These estimates are referred to as ‘pairwise’ dN/dS. We also calculated a lineage-specific estimate of dN/dS using the extremophile crucifer *Thellungiella parvula* (Dassanayake *et al*. 2011) as an outgroup, according to the method of Toll-Riera *et al*. (2011). First, we identified those *T. parvula* genes with detectable homology to an *A. thaliana* gene for >50% of the CDS length of the longest Col-0 transcript (blastn with default parameters). Multiple sequence alignments between the CDS of an *A. thaliana* gene, its *A. lyrata* orthologue (if extant) and the homologous sequence in *T. parvula* were made using PRANK (Löytynoja & Goldman 2008). dN/dS was calculated using the codeml package of PAML (Yang 2007), with the equilibrium codon frequencies of the model used as free parameters (CodonFreq = 3). These data were filtered to remove sequences less than 150 bp in length or with branches showing either dS < 0.02, dS > 2 or dN > 2 as these are either unreliable for estimates of the dN/dS ratio, nonbona fide orthologues or otherwise saturated with substitutions (Löytynoja & Goldman 2008). We assumed an unrooted tree topology of [(*A. thaliana*, *A. lyrata*), *T. parvula*].

The neutrality index for each sequence, NI, was calculated as log((2*D*_s_ + 1) (2*P*_n_ + 1)/(2*D*_n_ + 1) (2*P*_s_ + 1)), where *D*_s_ and *D*_n_ are the numbers of synonymous and non-synonymous substitutions, and *P*_s_ and *P*_n_ are the numbers of synonymous and non-synonymous polymorphisms (Haldane 1956). NI values can be tested with the null hypothesis of neutrality, that the ratios of intra- and interspecies non-synonymous to synonymous variation are equal. Positive selection is inferred when interspecies exceeds intraspecies variation – adaptive mutations spread throughout a population rapidly and so affect the number of observed substitutions (i.e. divergence), but not the number of polymorphisms (Egea *et al*. 2008). NI can thus be interpreted in the same manner as a McDonald–Kreitman test for comparing the ratio of fixed to within-species differences: its symmetrical distribution allows the inference of purifying selection when NI > 0 and positive selection when NI < 0 (McDonald & Kreitman 1991).

### Exon edge trimming

To assess the effect of exon edge conservation on rates of sequence evolution, we removed up to 30 codons from the edges of each *A. thaliana* exon that could be fully aligned against the *A. lyrata* or *T. parvula* genome with an alignment both in-frame and a multiple of three in length. Exons were then concatenated, and genes with sequences of at least 150 bp after trimming constituted ‘trimmed’ subsets of, at minimum, 1443 genes (i.e. those for which all 30 codons can be removed) and 174 genes, for alignments against *A. lyrata* and *T. parvula*, respectively. All analyses comparing ‘trimmed’ and ‘untrimmed’ sequences use the same set of exons per gene. A supplemental file containing both the raw alignments and evolutionary rate estimates for all data sets is available at the DRYAD repository (http://dx.doi.org/10.5061/dryad.905sq).

### Randomization test

Estimates of dN, dS, dN/dS and NI vary when codons are removed from the edges of exons, suggesting that the strength of selection differs in these regions. To assess whether the difference is indeed due to the nature of exon edges or due to codon removal, we created a parallel set of estimates of dN, dS, dN/dS and NI after random codon removal (*s* = 1000 randomizations per gene) for comparison. A numerical *P*-value was calculated as follows: letting *q* be the number of times the ‘sequential removal’ estimate of dN, dS or dN/dS was higher than the ‘random removal’ estimate (or lower, in the case of NI), then *p* = ((*s*-*q*) + 1)/*s* + 1. As variable estimates of dN, dS, dN/dS and NI can in turn alter the correlation strength with predictors of evolutionary rate (such as, e.g. expression level), the above test was also repeated using estimates of Spearman's *rho* for both the ‘sequential removal’ and ‘random removal’ conditions.

### Expression data

Three independent sources of *A. thaliana* transcript abundance data were used: (i) the Arabidopsis Development Atlas (ADA), representing 79 tissues, generated by the AtGenExpress Consortium (Schmid *et al*. 2005) [NASCARRAYS reference numbers 150–154, (http://affymetrix.arabidopsis.info/, downloaded 7 November 2011)]. Expression level was quantified both as the maximum absolute gcRMA [robust multi-array analysis corrected for the GC content of the oligo (Wu *et al*. 2004)] across all tissue types (after clustering the data into seven types – root, stem, seed, leaf, flower, pollen and apex) (Slotte *et al*. 2011) and as the average across all 79 tissues (Yang & Gaut 2011). Expression breadth was calculated from this database as both the number of tissues in which a gene is expressed and the tissue specificity index (*tau*), a scalar measure bounded between 0 (for housekeeping genes) and 1 (for genes expressed in a single tissue) (Yanai *et al*. 2005). (ii) Massive parallel signature sequencing (MPSS) data (Brenner *et al*. 2000; Meyers *et al*. 2004; Nakano *et al*. 2006) – which quantifies gene expression by counting short (17–20 bp) mRNA-derived tags – representing five tissues (http://mpss.udel.edu/at/mpss_index.php, downloaded 28 March 2011). Expression level was quantified as either the average (Yang 2009) or the maximum number of tags across all tissues (Foxe *et al*. 2008). (iii) RNA-seq transcript abundance data, where expression levels were taken as absolute read values corrected by sequence length (Gan *et al*. 2011). On top of the indices of expression obtained from each data set, all three estimates of transcript abundance (MPSS, ADA and RNA-seq) were transformed into *Z*-scores (Cheadle *et al*. 2003) to allow direct comparisons between them. In addition, the weighted average of two sets of *A. thaliana* protein abundance data was obtained for a total of 19 761 genes (pax-db.org, downloaded 15 February 2013) (Baerenfaller *et al*. 2008; Castellana *et al*. 2008). These data employ tandem mass spectrometry to quantify protein abundance by spectral counting.

### Alternative splicing

Alternative splicing indices were calculated as described in Chen *et al*. (2014). In brief, alternative splicing events were identified by comparing mapping coordinates from EST data [obtained from dbEST (Boguski *et al*. 1993); http://ftp://ftp.ncbi.nih.gov/repository/dbEST, downloaded 1 May 2011] to the genome sequence. To avoid biases introduced by differential transcript coverage between genes (Kim *et al*. 2007; Nilsen & Graveley 2010; Chen *et al*. 2012), we used a transcript number normalization method (Kim *et al*. 2007) whereby the number of alternative splicing events per gene is calculated as the average number of events detected using 100 random samples of 10 mapped ESTs.

### Correlations between evolutionary rates and functional and structural gene characteristics

To determine whether, and to what extent, any functional and structural variables affect a gene's dN/dS and NI estimates, various correlation analyses were performed. All analyses were conducted in R (R Development Core Team 2012). Initially, all correlations were assessed using Spearman's *rho*. However, as many of the variables found to be significantly associated with dN/dS are themselves covariates of expression level (the strongest correlate of dN/dS), it is possible that some parameters co-vary with dN/dS as a by-product of their relationship with expression level. As such, to better understand the relative contribution of genomic features to dN/dS, we assessed the relationship between individual parameters and dN/dS after controlling for the effect of expression level, using partial Spearman's correlation coefficients [R package ‘ppcor’ (Kim & Yi 2006, 2007)]. In addition, to test whether correlation strengths for dN/dS with any given genomic feature differ between pairwise and lineage-specific dN/dS estimates, we assessed statistical significance using a t-test on the Z-transformed values of *rho*, as implemented by the paired.r method of the R package ‘psych’ (Revelle 2014).

## Results

### Correlates of dN/dS and NI in *A. thaliana*

Expression level and breadth [calculated using RNA-seq data (Gan *et al*. 2011)] were significant predictors of dN/dS and NI (calculated from pairwise alignments between *A. thaliana* and *A. lyrata*) and were in fact their strongest predictors compared to other variables (Table 1 and Table S2 in Supporting information). Similar results were obtained when using independent expression-level estimates from two alternative platforms, microarrays and MPSS as well as when applying four normalization procedures previously used for each set of estimates (Table S2 in Supporting information).

**Table 1:** Correlation strength of dN/dS and NI with different variables in *A. thaliana*, after alignment against *A. lyrata*, *T. parvula* or both

	Alignments of *A. thaliana* with *A. lyrata*		Alignments of *A. thaliana* with *T. parvula*		Alignments of *A. thaliana* with both *A. lyrata* and *T. parvula*	
Variable	dN/dS	NI	dN/dS	NI	dN/dS	NI
Average exon length	0.103	−0.026	0.017	0.045	−0.141	−0.043
Average intron length	−0.070	0.043	−0.052	0.061	−0.027	0.088
Gene length	−0.243	0.092	−0.067	−0.047	−0.169	0.044
Primary transcript length	−0.243	0.092	−0.067	−0.047	−0.170	0.043
Protein length	−0.124	0.050	−0.015	−0.060	−0.186	−0.034
Total exon length	−0.203	0.075	−0.066	−0.039	−0.200	0.005
Total intron length	−0.228	0.086	−0.056	−0.041	−0.021	0.089
UTR length (5′)	−0.183	0.032	−0.131	0.003	−0.01	0.035
UTR length (3′)	−0.122	0.053	−0.070	0.040	−0.051	0.086
Expression breadth	−0.399	0.120	−0.284	0.117	−0.130	0.232
Exp. level (RNA-seq)	−0.415	0.145	−0.285	0.117	−0.143	0.217
Protein abundance	−0.302	0.078	−0.241	0.095	−0.086	0.194
Tissue specificity (*tau*)	0.277	−0.088	0.210	−0.092	0.128	−0.175
Effective number of codons	0.059	−0.016	0.065	−0.035	0.064	−0.043
Frequency of optimal codons	−0.194	0.065	−0.187	0.116	−0.069	0.176
GC (%)	−0.009	0.036	−0.057	0.081	−0.110	0.038
Intron density	−0.158	0.048	−0.022	−0.052	0.026	0.064
Total no. of introns	−0.212	0.071	−0.038	−0.069	−0.014	0.062
Multifunctionality	−0.132	−0.013	−0.137	−1.18 × 10^−4^	−0.045	−0.012
No. of protein–protein interactions	−0.060	0.031	−0.084	0.069	−0.113	0.152
Recombination rate	0.007	−0.058	−0.011	−0.019	0.026	−0.022

All values shown are correlation strengths, as Spearman's *rho*. All values are statistically significant at *P* < 0.05, except for those underlined.

When dN/dS and NI estimates were obtained from alignments of *A. thaliana* and *T. parvula*, a more distant relative of *A. thaliana* than *A. lyrata*, expression level and breadth remain the strongest predictors of both dN/dS and NI, albeit with comparatively weaker correlation strengths in the case of dN/dS variance (but equivalent correlation strengths for NI variance) (Table 1 and Table S2 in Supporting information). In general, other variables such as gene length and codon usage bias explain progressively smaller proportions of dN/dS and NI variance in an equivalent order to that using *A. thaliana*–*A.lyrata* estimates. The association between dN/dS and NI with any non-expression-related genomic parameter is not fully accounted for by its association with gene expression as after the effect of expression level is removed using partial Spearman's correlation coefficients, all significant correlates of dN/dS and NI remained so (Table 2 and Table S3 in Supporting information).

**Table 2:** Partial correlations of dN/dS and 11 evolutionary rate predictors in *A. thaliana*, after controlling for expression level

Variable	Alignments of *A. thaliana* with *A. lyrata*	Alignments of *A. thaliana* with *T. parvula*	Alignments of *A. thaliana* with both *A. lyrata* and *T. parvula*
Average exon length	0.077	0.008	−0.151
Average intron length	−0.037	−0.037	−0.014
Gene length	−0.155	−0.051	−0.156
Protein length	−0.093	−0.029	−0.196
Total exon length	−0.124	−0.052	−0.191
Total intron length	−0.148	−0.039	0.002
Total no. of introns	−0.136	−0.021	0.007
Frequency of optimal codons	−0.130	−0.121	−0.045
Expression breadth	−0.220	−0.148	−0.055
Protein abundance	−0.126	−0.112	−0.008
No. of protein–protein interactions	−0.108	−0.118	−0.118

All values shown are partial correlation strengths, as Spearman's *rho*. All values are statistically significant at *P* < 0.05, except those underlined.

### Accounting for exon edge conservation influences dN/dS and its relationship with various genomic parameters, and unmasks higher levels of positive selection

**Fig. 1:**
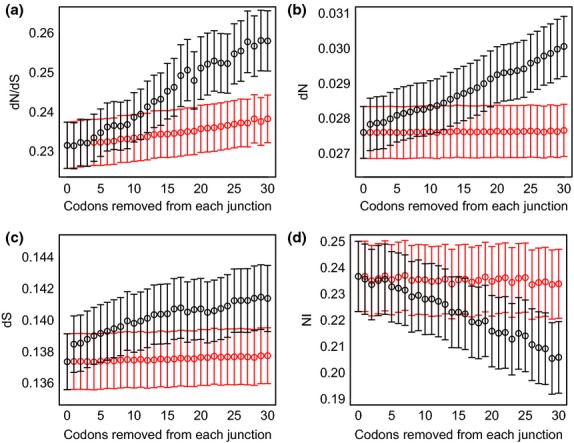
dN, dS, dN/dS and NI after exon edge removal. dN/dS (a), dN (b), dS (c) and NI (d) for a sample of 1443 genes with at least one fully alignable exon between *A. thaliana* and *A. lyrata*, after removing one codon at a time from exon edges (black), to a maximum of 30. The effects of random codon removal are shown in red. Distributions significantly differ when 30 codons are removed sequentially, but not randomly, compared to when no codons are removed. For sequential removal vs. no removal, Kruskal–Wallis *P* = 0.02 (dN/dS) and < 2.2 × 10^−16^ (NI). For random removal vs. no removal, Kruskal–Wallis *P* = 0.08 (dN/dS) and 0.49 (NI).

Using pairwise alignments of *A. thaliana* against either *A. lyrata* or *T. parvula*, we find that codon removal at the edges of exons results in increased dN, dS and dN/dS estimates when compared to estimates made after random codon removal from any position in the sequence (Fig.1 and Table S4 in Supporting information). This is observed irrespective of whether 10, 20 or 30 codons are removed (Table S4 in Supporting information). Estimates of NI were found to decrease after codon removal from the exon edges compared to random codon removal, also suggesting a weakening in the departure of sequence evolution from a neutral expectation (Fig.1 and Table S4 in Supporting information). These patterns are consistent with exon edges being under selective constraint, having fewer non-synonymous substitutions than sequence elsewhere in the gene. In general, exon edge removal shifts dN/dS values towards a range indicative of either stronger positive or relaxed purifying selection, with an overall increase in the proportion of genes potentially under adaptive selection (Table 3 and Table S5 in Supporting information).

**Table 3:** Exon edge removal shifts dN/dS values towards a range indicative of either stronger positive or relaxed purifying selection, with the proportion of genes potentially under adaptive selection increased

						Chi-square test	
Dataset	Max. no. of codons removed from each gene	No. of genes	% of genes with dN/dS >1 (no codons removed)	% of genes with dN/dS >1 (after sequential codon removal)	% of genes with dN/dS >1 (after random codon removal)	χ^2^	*P*
Alignments of *A. thaliana* against *A. lyrata*	10	3213	1.81	2.4	1.81	11.25	7.96 × 10^−4^
	20	2041	1.62	2.45	1.71	6.43	0.011
	30	1443	1.39	2.43	1.39	6.22	0.013
Alignments of *A. thaliana* against *T. parvula*	10	779	0.64	1.67	0.77	8.17	4.27 × 10^−3^
	20	350	0.29	1.43	0.29	16.00	6.33 × 10^−5^
	30	174	0	2.87	0	NA	NA

**Fig. 2:**
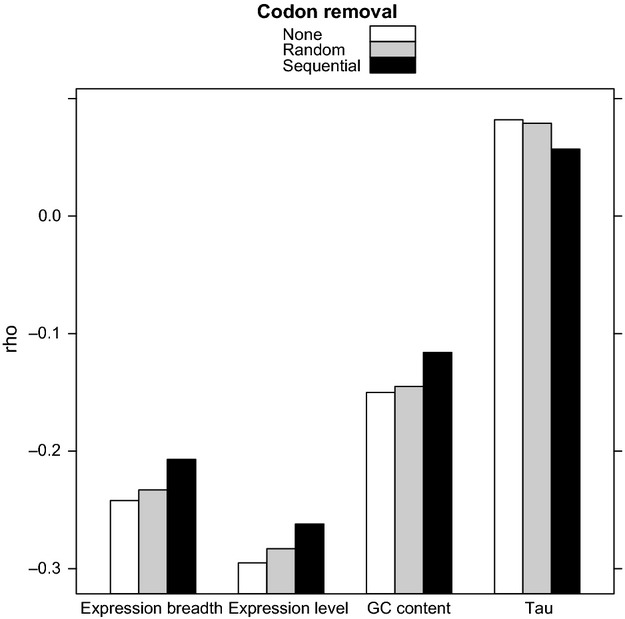
Variables that have a significantly different correlation with dN/dS after the sequential removal of 30 codons from exon edges, compared to random codon removal. The four variables shown – expression breadth, expression level, *tau* and GC content – are those which have significantly different estimates of *rho* for their correlation with dN/dS before and after codon removal. Two criteria are met for each variable: that *rho* is significantly different after sequential, compared to random codon removal, and that *rho* is significantly different after sequential, compared to no codon removal. Estimates of dN/dS are made using alignments of *A. thaliana* against *A.lyrata*. Data for this figure, including *P*-values and sample sizes, are shown in Table S6 (Supporting information).

To understand the effect of higher conservation at the exon edges on the relationships between dN/dS and other genomic parameters, we then re-analysed the correlations. We found that the correlation strength of dN/dS with several genomic features – in particular, expression level and expression breadth – decreased after the removal of exon edges. In contrast, we observed only marginal changes to these correlation coefficients after removing an equivalent number of codons from random positions (Fig.2 and Table S6 in Supporting information). This suggests that, after the removal of exon edges, the decreased correlation strength between dN/dS and genomic parameters is not explained by increased noisiness resulting from the use of shorter sequences to estimate dN/dS. It also suggests that a dN/dS-based test of selection is most acute for more highly expressed genes and that stronger correlations of dN/dS with their various characteristics reflect the stronger constraints upon them. Furthermore, when considering NI, several variables including expression level, expression breadth, the total number of introns and various measures of gene length become marginally, but significantly, stronger predictors of NI (Table S6 in Supporting information). Nevertheless, the relative order of these parameters as predictors of dN/dS remains largely unchanged with expression level still the dominant predictor.

### Reduced prominence of gene expression as a predictor of *A. thaliana's* lineage-specific dN/dS

Lineage-specific dN/dS estimates derived from multiple alignments of *A. thaliana* genes with *A. lyrata* and *T. parvula* resulted in a marked decrease in the correlation between dN/dS and various genomic parameters including expression level and expression breadth, with total exon length becoming the strongest correlate of dN/dS (Table 1 and Table S2 in Supporting information).

To rule out the possibility that reductions in both the absolute and relative strength of the correlation between dN/dS and gene expression when examining lineage-specific changes may be explained by differences in the gene/codon set tested, we recalculated pairwise dN/dS for *A. thaliana* against *A. lyrata* and *T. parvula* using only those codons common to the multiple alignments of *A. thaliana*, *A. lyrata* and *T. parvula* (i.e. those used to estimate lineage-specific dN/dS; Table S7 in Supporting information). This analysis confirmed that when the same codons are analysed, lineage-specific dN/dS estimates have markedly weaker correlations with numerous genomic features compared to either pairwise estimate (Table 4 and Table S8 in Supporting information).

**Table 4:** Correlates of dN/dS using estimates derived from codons common to the alignment of *A. thaliana*, *A. lyrata* and *T. parvula*

Variable	Alignments of *A. thaliana* with *A. lyrata*	Alignments of *A. thaliana* with *T. parvula*	Alignments of *A. thaliana* with both *A. lyrata* and *T. parvula*
Average exon length†	−0.040	−0.040	−0.106
Average intron length	−0.080	−0.060	−0.002
Gene length	−0.146	−0.115	−0.154
Primary transcript length	−0.146	−0.115	−0.154
Protein length†	−0.107	−0.089	−0.177
Total exon length	−0.139	−0.114	−0.182
Total intron length	−0.088	−0.072	−0.040
UTR length (5′)	−0.012	−0.007	0.045
UTR length (3′)	−0.066	−0.056	−0.017
Expression breadth†	−0.286	−0.317	−0.182
Exp. level (RNA-seq)†	−0.256	−0.284	−0.144
Protein abundance†	−0.198	−0.225	−0.102
*Tau* (tissue specificity)†	0.214	0.239	0.124
Effective number of codons†	0.116	0.123	0.051
Frequency of optimal codons†	−0.142	−0.189	−0.051
GC (%)	−0.054	−0.076	−0.087
Intron density	−0.050	−0.056	−0.020
Total no. of introns	−0.079	−0.067	−0.045
Multifunctionality	−0.060	−0.038	−0.036
Protein–protein interactions	−0.127	−0.153	−0.098
Recombination rate	−0.001	−0.063	0.041

Correlation strengths are shown as Spearman's *rho*. All values are statistically significant at *P* < 0.05, except those underlined. The rightmost column shows lineage-specific dN/dS estimates.

† Significantly different correlation strength when using lineage-specific dN/dS estimates compared to pairwise estimates.

We further found that using lineage-specific substitution patterns markedly reduces the number of genes with dN/dS >1 (21 genes have dN/dS >1, 0.3% of the sample analysed) when compared to pairwise alignments of *A. thaliana* with *A. lyrata* (423 genes have dN/dS >1, 2% of the sample analysed; chi-square *P* < 2.2 × 10^−16^), although not when compared to alignments with *T. parvula* (41 genes have dN/dS > 1, 0.4% of the sample analysed, chi-square *P* = 0.327). In summary, when examining lineage-specific dN/dS estimates, the prominence of gene expression is diminished, and protein length becomes the dominant predictor. This pattern is not explained by variations in the sample of genes/codons used for the analyses. Importantly, we observed no evidence that the use of lineage-specific dN/dS estimates unmasks any additional signatures of positive selection compared to pairwise alignments.

## Discussion

### Selective constraint upon exon edges affects the relationship between dN/dS and expression

Previous studies have shown that in mammalian species, exonic splicing enhancer sequences result in higher conservation of synonymous sites at exon edges, suggestive of selective constraint to maintain correct splicing (Carlini & Genut 2006; Parmley *et al*. 2006). Here, we show that the removal of codons at the exon edges has a strong effect on the rate of substitutions at synonymous sites in *A. thaliana*, suggesting similar constraint, and associated functional importance, for ESE-containing regions in plants. A moderate increase was also observed in the rate of non-synonymous substitutions reflecting the fact that purifying selection at these sites is higher than the average observed at non-synonymous sites elsewhere in the gene.

This study is, to the best of our knowledge, the first to explore the relationship between rates of sequence evolution and genomic parameters (including gene expression) in the context of exon edge conservation due to the presence of splice enhancers. Generally, the removal of exon edges resulted in a weaker association between dN/dS and NI with measures of expression level and breadth. The relationship between dN/dS and other genomic parameters – such as various measures of gene/protein length – showed a moderate decrease, whereas the association between several length parameters and NI was strengthened (Table S6 in Supporting information). The observed decrease in the relationship between dN/dS and NI to gene expression after the removal of exon edges suggests that a stronger degree of purifying selection acting upon splice enhancer regions partly explains the association between dN/dS, NI and expression. From this, we can infer stronger splice-mediated selection in more highly expressed genes.

It is possible that more highly expressed genes are under increased constraint for accurate splice site definition, with this relationship partly masked by the stronger association of higher expression with lower dN/dS, which largely reflects constraint on the gene's function. In this respect, selection may also be masked on other properties expected to be under stronger constraint in more highly expressed genes, such as codon usage affecting translational error rate (Drummond *et al*. 2005), translation efficiency (Akashi & Eyre-Walker 1998) and mRNA stability (Tuller *et al*. 2010), although such analyses are beyond the scope of this study.

It is reasonable to ask whether anything can explain the higher selective constraint upon exon edges in such a way as to also relate both to a gene's structure and to its expression. One possible explanation may be the extent to which a gene is alternatively spliced. Alternative splicing has been shown to positively correlate with both the ratio of total intron length to overall gene length (Koralewski & Krutovsky 2011) and gene expression level (Chen *et al*. 2014). As longer genes are more likely to have more complex exon–intron architectures (Zhu *et al*. 2009), they are expected to have a higher number of possible alternative splicing events. If we assume that the exon edges are under increased selection for accurate alternative splicing compared to non-alternatively spliced exons, then those genes with higher levels of alternative splicing are expected to show a greater discrepancy in evolutionary rate estimates before and after codon removal. Using estimates of the number of alternative splicing events per gene, we find that dN/dS ratios (calculated from pairwise alignments of *A. thaliana* and *A. lyrata* to maximize sample size) are more strongly affected by codon removal from the exon edges in genes with higher levels of alternative splicing – for instance, the increase in dN after 10 codons are removed is significantly higher for genes with more splicing events (*rho* = 0.13, *P* = 2.7 × 10^−4^; Table S9 in Supporting information). This pattern is also observed when removing 20 or 30 codons from exon edges (Table S9 in Supporting information). Although based upon a limited sample size, this finding merits further scrutiny as it shows that genes with alternative splicing events, compared to nonspliced genes, have a higher degree of conservation at exon edges relative to conservation of the remaining coding sequence.

### Lineage-specific dN/dS estimates have a stronger relationship with gene length than with expression level

The use of pairwise alignments for estimating dN/dS could influence any relationship between dN/dS and a gene's characteristics as biases are introduced due to branch-specific changes in the strength and direction of selection. For example, if a gene in *A. lyrata* was under a greater degree of purifying selection than its *A. thaliana* orthologue, this would result in a decreased dN/dS estimate in *A. thaliana* (Toll-Riera *et al*. 2011). This would introduce noise into the correlation of dN/dS and any genic feature in *A. thaliana*. Estimating a lineage-specific dN/dS using *T. parvula* as an outgroup, we found the correlation strength of dN/dS with many genic features, both structural and functional, is reduced (Table S2 in Supporting information). In particular, the estimate of *rho* for the expression level–dN/dS relationship is reduced by more than 50% when using a lineage-specific compared to a pairwise dN/dS estimate (Table 1 and Table S2 in Supporting information). However, the use of lineage-specific dN/dS estimates increased the correlation between dN/dS and gene length. This is of interest given the relationship between the three variables – as expression and length are both negative correlates of dN/dS, it follows that genes under stronger purifying selection are more likely to be both highly expressed and shorter. As selection for higher expression can reasonably predict a gene's length, with shorter genes minimizing costly transcription and translation (Castillo-Davis *et al*. 2002; Eisenberg & Levanon 2003; Urrutia & Hurst 2003), this suggests that gene length itself, rather than expression, could be a stronger predictor of dN/dS. This finding also supports a previously observed negative relationship between dN/dS and gene length identified using *A. thaliana*–*A. lyrata* orthologous pairs (Yang & Gaut 2011).

It is possible that the comparatively reduced prominence of expression level as a predictor of evolutionary rate is explained in this case by mating system: *A. thaliana*, unlike *A. lyrata* or *T. parvula*, is a near obligate selfer, having a patchy distribution of inbred populations with relatively rare outcrossed mating between different ecotypes (Tian *et al*. 2002). Selfing increases genomewide homozygosity, and thus decreases the number of gametes which may be independently sampled in a given population, in effect reducing effective population size (Szövényi *et al*. 2014). As a consequence, the efficacy of selection – particularly purifying selection – at purging weakly deleterious mutations is reduced (Wright *et al*. 2013; Glemin & Muyle 2014). In this respect, the degree of constraint acting upon highly expressed genes may be partially masked when using lineage-specific dN/dS estimates. Nevertheless, that *A. thaliana* experiences a general trend of relaxed selection compared to *A. lyrata* is only weakly supported (Glémin 2007) and in any case, the relationship of expression level to lineage-specific dN/dS for *A. lyrata* is equally reduced, assuming expression to be equivalent in both species (*rho* = −0.15, *P* < 2.2 × 10^−16^; Table S2 in Supporting information). In addition, it is important to note that the differences between pairwise and lineage-specific dN/dS are not explained by the differences in gene/codon samples used to estimate dN/dS resulting from the fact that a smaller proportion of the *A. thaliana* genome can be simultaneously aligned with both the *A. lyrata* and the *T. parvula* genomes as similar results are obtained when restricting the analyses to a common set of codons. Finally, we believe that *T. parvula* is a justifiable outgroup species as it has an estimated divergence time from *A. thaliana* of approx. 40 mya; this falls within the range of distances for species used to calculate lineage-specific dN/dS (e.g. approx. 90 mya for the divergence of humans and dogs, as in Toll-Riera *et al*. (2011)) without confounding the estimate by saturation.

### Exon edge removal, but not lineage-specific substitution patterns, unmasks higher levels of positive selection

One key objective of this study was to assess whether exon edge conservation and the use of pairwise alignments could be masking higher levels of molecular adaptation than what has previously been observed. In general, we find that the proportion of genes under potential positive selection (dN/dS >1) is increased by the removal of exon edges. Of particular interest are four genes (AT1G08680, AT1G60930, AT2G17305 and AT4G27370) where dN/dS ratios are higher than 1 only after codons are removed from the exon edges, but not when codons are removed from random positions. This could suggest, in these cases, that an adaptive signature has been partially masked by disproportionate synonymous substitutions at the edges of exons. Of note is that AT1G08680 (ARF GAP-like zinc finger-containing protein ZIGA4) has been linked to adaptive germination phenotypes (Morrison & Linder 2014) and that AT1G60930 (RECQ helicase L4B) appears to be a duplicate gene that has undergone a degree of functional divergence (Singh *et al*. 2010). As duplicated genes undergo asymmetric sequence divergence relative to each other (Conant & Wagner 2003), an adaptive interpretation is in this case plausible.

When considering lineage-specific dN/dS, however, the proportion of genes with dN/dS >1 is significantly lower than when dN/dS is estimated using pairwise alignments of *A. thaliana* with *A. lyrata*. This could indicate that dN/dS values higher than 1 are, for several genes, being driven by increased dN/dS values in the *A. lyrata* lineage which, notably, does not show prevalent self-fertilization.

Having found a significant effect of exon edge conservation and lineage-specific substitution upon dN/dS estimates when each was considered separately, we wished to test whether the relationship between dN/dS and the set of genomic parameters changed when both factors are taken into account together. However, there were only a limited number of genes for which full exons could be aligned across all three species, as required for the analysis of codon removal at the exon edges and the estimates of lineage-specific dN/dS. Using a limited sample (*n* = 73) in which 10 codons could be removed from the exon edges, we found no significant differences in the relationship of dN/dS to any genomic parameter after codons were removed from the exon edges compared to removal at random sites (Table S6 in Supporting information). Better annotation of *A. lyrata* and *T. parvula*, or the genomes of related species, would improve the testing of the effects of exon edge conservation upon dN/dS estimates using lineage-specific substitutions.

The variation in sequence evolution among genes and its association with genic characteristics, including expression, could also be partly explained by genomic context. Most notably, chromosomal location has been associated with gene expression in *A. thaliana* (Yamada *et al*. 2003; Schmid *et al*. 2005). Several studies have also shown that across the genome, there are nonrandom clusters of genes with similar expression profiles in a variety of taxa (Lercher *et al*. 2002; Versteeg *et al*. 2003). Clusters of genes with similar evolutionary rate have also been identified (Williams & Hurst 2000; Lercher *et al*. 2001). A common mechanism may explain both clusters (i.e. Williams & Hurst (2002) but see Lercher *et al*. (2004)), although further assessment of such hypotheses fall outside of the scope of this study.

In summary, we show that higher conservation at the edges of exons in *A. thaliana* plays an important part in determining dN/dS ratios by increasing the proportion of conserved synonymous sites. The effect of these conserved regions upon overall dN/dS values partly explains the relationship between rates of protein evolution and expression level. By accounting for lineage-specific substitution patterns and the effect of conservation at the exon edges, the ability of expression level to explain variation in evolutionary rate is diminished, with gene length becoming the strongest correlate. In addition, we found evidence of masked positive selection from the conservation of exon edges, irrespective of the noise introduced to dN/dS estimates by the use of pairwise alignments.

## Data accessibility

Alignments and associated evolutionary rate estimates are available for download at the DRYAD repository (http://datadryad.org), entry doi:10.5061/dryad.905sq.

## Supporting information

**Table S1** Structural and functional characteristics of *A. thaliana* genes. **Table S2** Relationship between dN/dS and NI with various genomic characteristics in *A. thaliana*. **Table S3** Partial correlations between dN/dS and 20 genomic characteristics in *A. thaliana*, controlling for expression level. **Table S4** Average estimates of four evolutionary rate variables after sequential codon removal from the exon edges vs. random codon removal. **Table S5** Characteristics of the dN/dS and NI distributions for dataset A (pairwise alignment of *A. thaliana* with *A. lyrata*) and dataset B (pairwise alignment of *A. thaliana* with *T. parvula*), before and after codon removal at exon-intron junctions. **Table S6** Correlations between 4 selection strength/direction variables and 25 genomic characteristics, after the removal of 10, 20 and 30 codons from the exon edges vs. random removal of an equal number of codons. **Table S7** dN/dS estimates using codons common to the alignment of *A. thaliana*, *A. lyrata* and *T. parvula*. **Table S8** Relationship between dN/dS and evolutionary rate predictors using estimates derived from codons common to the alignment of *A. thaliana*, *A. lyrata* and *T. parvula*. **Table S9** Relationship between the average number of alternative splicing events per gene and the difference in evolutionary rate estimates before and after codon removal from the exon edges.
